# Torsion of an Accessory Spleen in a Child With Biliary Atresia Splenic Malformation Syndrome

**DOI:** 10.3389/fped.2020.00220

**Published:** 2020-05-04

**Authors:** David A. Simon, Nathan R. Fleishman, Pamala Choi, Jason D. Fraser, Ryan T. Fischer

**Affiliations:** ^1^Department of Pediatrics, Children's Mercy Hospitals and Clinics, Kansas City, MO, United States; ^2^Department of Gastroenterology and Hepatology, Children's Mercy Hospitals and Clinics, Kansas City, MO, United States; ^3^Department of Pediatric Surgery, Children's Mercy Hospitals and Clinics, Kansas City, MO, United States

**Keywords:** accessory spleen, torsion, biliary atresia splenic malformation syndrome (BASM), abdominal pain, congenital anomaly

## Abstract

Torsion of an accessory spleen is an exceedingly rare cause of abdominal pain in pediatric patients. The diagnosis is frequently challenging as presentation is variable and diagnostic imaging can be aspecific. The current case describes an unusual presentation of a torted accessory spleen in a 5-year-old girl with biliary atresia splenic malformation syndrome who initially presented with non-specific abdominal symptoms and fever. The diagnosis was made following fine-needle aspiration of a suspected intraabdominal abscess. The case highlights the diagnostic challenge of accessory splenic torsion and stresses the importance of its inclusion on the differential diagnosis of pediatric patients, especially those with known splenic or laterality abnormalities, presenting with both acute and sub-acute abdominal symptoms.

## Background

Accessory spleens or splenules represent a congenital focus of normal splenic tissue separate from the spleen. They are typically asymptomatic and observed in ~10–30% of patients at autopsy (1, 2). Rarely, accessory spleens become symptomatic following torsion of their vascular pedicle, which compromises blood flow to the organ, leading to a potentially wide spectrum of clinical presentations. Cases may present with a variety of non-specific symptoms (e.g., vague abdominal pain, nausea, vomiting, and fever), recurrent abdominal pain, or with an acute abdomen (3). Diagnosis is typically made in the operating room as diagnostic imaging is often times unrevealing. Definitive treatment is almost always splenectomy (4, 5). Herein, the authors describe an unusual presentation of a torted splenule in a 5-year-old patient with biliary atresia splenic malformation syndrome (BASM).

## Case Presentation

A 5-year-old girl was admitted with a 1-day history of generalized malaise, right upper quadrant abdominal pain, vomiting, and fever. Her medical history is significant for mosaic terminal 18q deletion, bronchomalacia, and BASM (with known situs inversus, polysplenia, and malrotation) status post hepatoportoenterostomy with resulting chronic liver fibrosis.

She was initially well appearing with a benign abdominal examination and normal vital signs. Laboratory evaluation including basic metabolic panel, complete blood count with differential, prothrombin time/international normalized ratio, and hepatic function panel were reassuring. Blood cultures were obtained and piperacillin/tazobactam was initiated secondary to concern for possible cholangitis. Metronidazole was added after stool pathogen panel returned positive for the giardia antigen. Interestingly, abdominal x-ray, obtained around time of admission, showed significant colonic stool burden, and a continuous polyethylene glycol infusion via nasogastric tube was run for 24 h with good effect in terms of stool output and relief of abdominal pain.

On day 3 of admission, despite broad spectrum antibiotics and negative blood cultures, the patient continued to be febrile and developed marked abdominal distension. Abdominal ultrasound was obtained and demonstrated perihepatic and lower abdominal ascites. Right-sided spleen was visualized with normal parenchymal echotexture. Ultrasound guided paracentesis was performed, with fluid analysis revealing 3,724 total nucleated cells with 71% neutrophils. Peritoneal fluid culture did not speciate an organism, however, at the time, this was believed to be due to antibiotic sterilization. A presumptive diagnosis of spontaneous bacterial peritonitis was made, and after consultation with Infectious Diseases, intravenous ceftriaxone was initiated for better empiric coverage of encapsulated organisms.

**Figure 1:**
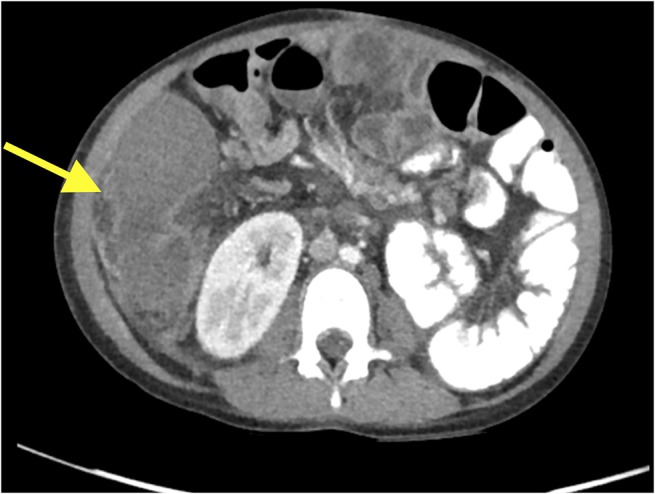
Axial CT scan demonstrating a right intra-abdominal rim enhancing lesion.

**Figure 2:**
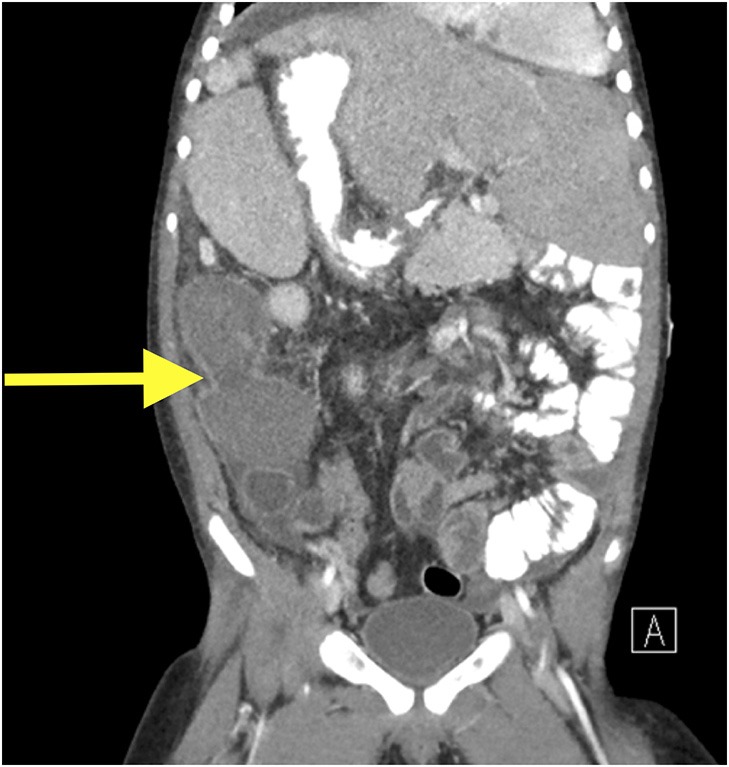
Coronal CT scan demonstrating a right intra-abdominal rim enhancing lesion.

**Figure 3:**
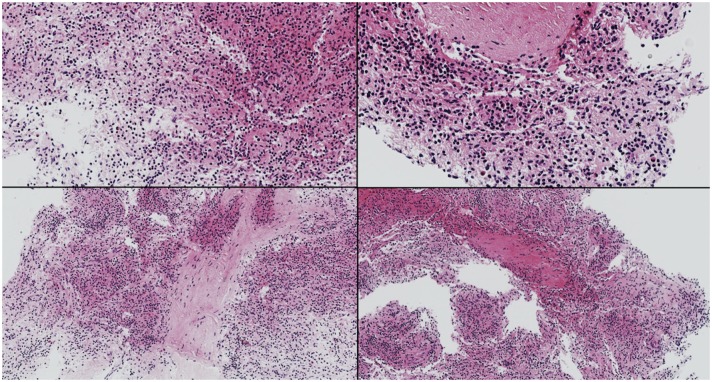
Fragments of splenic tissue with patchy hemorrhagic necrosis consistent with splenic infarction.

Despite greater than 48 h of appropriate antibiotics, the patient continued to be intermittently febrile and complain of significant abdominal discomfort and distension. Subsequent abdominal computed tomography (CT) showed a large, rim enhancing fluid collection in the right upper abdomen concerning for abscess (see Figures 1, 2). After consultation with Pediatric Surgery, the patient was taken to Interventional Radiology for abscess drainage. Upon attempted fluid aspiration, soft tissue was obtained. Pathological evaluation revealed the tissue to be consistent with a splenic infarction (see Figure 3). Given the location and appearance of the tissue, a diagnosis of accessory splenic torsion was made.

**Figure 4:**
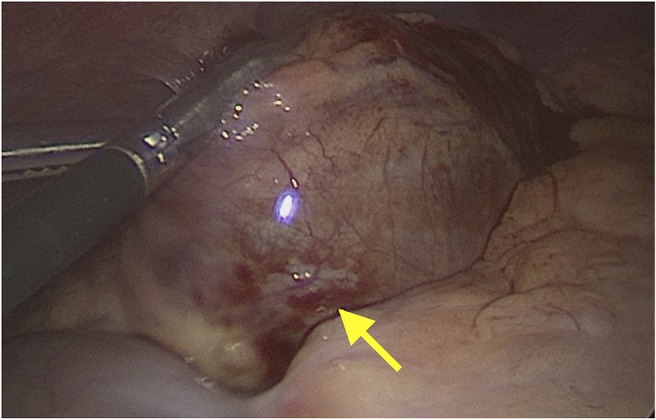
Laparoscopic image showing congestion and ischemia of the accessory spleen *in situ*.

The initial treatment approach included conservative management with antibiotics, nasogastric tube feedings and pain control based on the desire to avoid surgical intervention in a patient with a complex medical and surgical history, as well as the noted resolution of splenic infarction without surgery (6). However, due to persistent fevers and uncontrolled abdominal pain, the patient ultimately underwent removal of the infarcted splenule via laparoscopic assisted excision (see Figure 4). Surgery was well-tolerated, and histological examination confirmed the diagnosis of accessory splenic torsion. Post-operative course was uneventful, and the patient was discharged from the hospital on post-operative day 8.

## Discussion

The human spleen is derived from proliferating mesenchyme overlying the dorsal pancreatic endoderm between the tiers of the dorsal mesogastrium during the fifth week of embryogenesis (1). Between weeks 7 and 8 the rotation of the stomach causes the left mesogastrium surface to fuse with the peritoneum above the left kidney. An accessory spleen is formed when mesenchymal remnants fail to fuse with the main splenic mass and deposit along the path of splenic development (1). They are exclusively found on the left side of the abdomen, however in the case of situs inversus (as seen in the current case), right sided accessory spleens are observed (5).

Accessory spleens are a frequently observed congenital anomaly occurring in ~10–30% of patients on postmortem studies (1, 2). Of those with accessory spleens only 14% have more than one (7). Accessory spleens are almost always asymptomatic. Complications, such as torsion and infarction, are extremely rare. To the authors knowledge, only 19 pediatric cases of accessory splenic torsion, including the current case, have been described in the English literature (2–6, 8–18). This is the second reported case of an accessory splenic torsion occurring in a child with known situs inversus and the only documented case in a patient with BASM (5).

Biliary atresia describes a progressive, idiopathic, fibro-obliterative disease of the extrahepatic biliary tree and is the most common indication for liver transplantation in pediatric patients. Biliary atresia often presents in isolation; however, the condition can present in combination with congenital malformations. In the setting of known splenic abnormalities the condition is referred to as BASM. BASM occurs in ~10–15% of patients with biliary atresia (19, 20). In addition to splenic malformations (e.g., polysplenia or asplenia), situs inversus, malrotation, interrupted inferior vena cava, and cardiac anomalies are characteristic of the condition. Retrospective data suggests that patients with BASM have poorer outcomes than children with biliary atresia without other anomalies or malformations (21). These differences appear to be related to the higher risk of developing hepatorenal syndrome as well as various cardiac and intrabdominal complications. There are no known risk factors for developing an accessory splenic torsion, however it remains possible that pediatric patients with known splenic and intrabdominal abnormalities could be predisposed to this rare complication.

Diagnosis of a torted accessory spleen can be challenging, and separating the findings from possible cholangitis, gastroenteritis, bacterial peritonitis, abdominal abscess, and constipation in the above case proved difficult. Maintaining a high index of suspicion, especially in a patient with known splenic anomalies, is paramount as results of diagnostic imaging may be non-specific or unavailable in the emergency setting. As seen in Figures 1, 2, CT appearance of an accessory splenic torsion may mimic a drainable abdominal fluid collection or abscess (8). Ultrasonography with Doppler and magnetic resonance imaging may allow for further characterization of a possible torted splenule, however even with modern imaging modalities the diagnosis is often uncertain.

Almost all reported pediatric cases of accessory splenic torsion are diagnosed at time of surgery (4, 5, 9, 11, 13, 14, 16–18). Once confirmed, management is typically operative, however conservative management has proven successful (6). Scire et al. (6) report a 10-year-old male patient diagnosed with accessory splenic torsion after presenting to the emergency room with acute abdominal pain. He was treated with analgesics and antibiotics and had spontaneous symptom resolution (e.g., abdominal pain) within 1 week. Subsequent ultrasound imaging revealed progressive reduction in the dimensions of the accessory spleen at 3-month, 6-month, and 1-year follow-ups. Unfortunately, despite a similar approach, conservative management in the current case proved unsuccessful and surgical intervention was ultimately required.

## Conclusion

Accessory splenic torsion is a rare pediatric entity and should remain on the differential for pediatric patients presenting with both acute and non-specific abdomen pain, especially in the setting of known splenic malformations and/or other laterality defects. Once confirmed, conservative management could be considered, but operative management is most common.

## Data Availability Statement

All datasets analyzed for this study are included in the manuscript.

## Ethics Statement

Signed informed consent was obtained from the patient's mother for publication of this case report and any potentially identifying information has been removed.

## Author Contributions

DS, NF, RF, PC, and JF conceptualized, drafted, reviewed, and revised the manuscript. All authors approved the final manuscript as submitted and agree to be accountable to all aspects of the work.

## Conflict of Interest

The authors declare that the research was conducted in the absence of any commercial or financial relationships that could be construed as a potential conflict of interest.

## Abbreviations

BASM: Biliary atresia splenic malformation syndrome.
